# Restraining the Proliferation of Acute Lymphoblastic Leukemia Cells by Genistein through Up-regulation of B-cell Translocation Gene-3 at Transcription Level

**DOI:** 10.31661/gmj.v8i0.1229

**Published:** 2019-03-26

**Authors:** Masoumeh Abedi Nejad, Mohsen Nikbakht, Masoomeh Afsa, Kianoosh Malekzadeh

**Affiliations:** ^1^Department of Biology, Jahrom Branch, Islamic Azad University, Jahrom, Iran; ^2^Molecular Medicine Research Center, Hormozgan Health Institute, Hormozgan University of Medical Sciences, Bandar Abbas, Iran; ^3^Hematology-Oncology and Stem Cell Transplantation Research Center, Tehran University of Medical Sciences, Tehran, Iran; ^4^Department of Medical Genetics, Faculty of Medicine, Hormozgan University of Medical Sciences, Bandar Abbas, Iran

**Keywords:** Acute Lymphoblastic Leukemia, Anti-Proliferation, B-cell Translocation Gene-3, Genistein

## Abstract

**Background:**

Acute lymphoblastic leukemia (ALL) is a highly prevalent pediatric cancer accounting for approximately 78% of leukemia cases in patients younger than 15 years old. Different studies have demonstrated that B-cell translocation gene 3 (*BTG3*) plays a suppressive role in the progress of different cancers. Genistein is considered a natural and biocompatible compound and a new anti-cancer agent. In this study, we evaluate the effect of genistein on *BTG3* expression and proliferation of ALL cancer cells.

**Materials and Methods:**

ALL cell lines (MOLT4, MOLT17, and JURKAT) were cultured in standard conditions. Cytotoxicity of genistein was detected using MTT assay. The cells were treated with different concentrations of genistein (10, 25, 40, and 55μM) for 24, 48, and 72 hours, and then cell viability and growth rate were measured. The quantitative real-time polymerase chain reaction was applied to investigate the effect of genistein on *BTG3* expression.

**Results:**

The percentage of vital cells treated with genistein significantly decreased compared to the non-treated cells, showed an inverse relationship with an increasing genistein concentration. The present study suggests a dose of 40μM for genistein as a potent anticancer effect. Genistein could elevate *BTG3* for 1.7 folds in MOLT4 and JURKAT and 2.7 folds in MOLT17 cell lines at transcription level conveged with 60 to 90% reduction in the proliferation rate of cancer cells.

**Conclusion:**

Up-regulation of *BTG3* as a tumor suppressor gene can be induced by genistein. It seems that *BTG3* reactivation can be introduced as another mechanism of anti-proliferative effect of genistein and could be considered as a retardant agent candidate against hematopoietic malignancy.

## Introduction

Acute lymphoblastic leukemia (ALL) is the main pediatric cancer in developed countries, and the prevalence rate of the disease is approximately 30 to 45 cases in one million children per year [1]. ALL is a multifactorial disease promoted by exogenous or endogenous exposure and genetic susceptibility [2]. ALL originates from B- or T-lineage lymphoid precursors (B-ALL or T-ALL). The T-cell type accounts for approximately 15 to 25% of ALL diagnosed patients [3]. Recent investigations have demonstrated that the existence of the T-cell type of ALL is associated with a lesser post-recurrence survival [4]. Therefore, further investigations are required for this group of ALL. New DNA technologies such as microarray and next-generation sequencing findings of ALL have shed light on the molecular basis of this disease. These discoveries have shown that aberrant changes in genetic and epigenetic in genes such as tumor suppressors can disturb main cellular pathways. As some of the pathways, the changes in epigenetic and aberrant in kinase-activating lesions are rational targets for new medicines [5]. During the past few years, a wide spectrum of tumor suppressor genes has been demonstrated to be mutated and deleted in T-ALL [6]. Tumorigenesis is a multistep process occurring as a result of genetic and epigenetic alterations [7]. B-cell translocation gene 3 (*BTG3*), also known as *ANA*, *TOB5*, *TOFA*, *APRO4*, *TOB55*, and *ANA/BTG3*, belongs to the BTG gene family and plays its role as a downstream target of p53[8-9]. *BTG3* has a negative regulatory effect on cellular S-phase progression through interaction with transcription factor *E2F1* [10-11]. Recent investigations have indicated that *BTG3* acts as a tumor suppressor against the progression of cancer and loss of *BTG3* expression is frequently accompanied by tumor development in several different cancers [11-18]. In some human cancers, abnormal hypermethylation or histone modification in promotor of *BTG3* has been reported [11, 16, 19, 20]. Genistein (4ʹ,5,7-trihydroxyisoflavone) is a natural phytoestrogen found in soybeans and is one of the compounds that has recently attracted considerable attention in order to prevent and treat cancer. Some investigations have demonstrated the inhibitory actions of genistein in human breast cancer [21-23]. Another study has confirmed the effect of genistein on growth retardation in renal cell carcinoma [11]. One research has indicated that down-regulation of *BTG3* can be reactivated by genistein in prostate cancer [16]. To the best of our knowledge, there is no well-documented study regarding the *BTG3* expression profile in ALL. Also, the impact of genistein on *BTG3* expression has not been investigated so far. Therefore, we attempted to address this issue in the present study.

## Materials and Methods

### Samples and Cell Culture

Three ALL cell lines (MOLT17, MOLT4, and JURKAT) were obtained from the Pasteur Institute, Iran. Cell lines were cultured in RPMI-1640 (Gibco; Germany), medium supplemented with 10% fetal bovine serum (FBS; Sigma-Aldrich, Germany), and 500μl penestrep (penicillin and streptomycin; Invitrogen, Germany) in a humidified condition with 5% CO_2_ at 37^0^C. The cells were separated every 4-6 days. Then, neobar lam and trypan blue were used to count cells and determine cell viability, respectively.

### Toxicity Assay

The effect of genistein (Merk, Germany) on cellular proliferation was analyzed using 3-(4,5-dimethylthiazol-2-yl)-2,5-diphenyltetrazolium bromide MTT (Sigma; USA) assay based on standard protocols. First, 5×10^5^cells were seeded in 96-well plates and incubated for 3-4 hours. Then, they were treated with various genistein doses (10, 25, 40, and 55μM). Furthermore, one lane as a negative control and without treatment was included. The MTT survival assay was then conducted at different period times (24, 48, and 72 h). Cell viability was predicted via a colorimetric analysis and converting tetrazolium dye (MTT) to a product of blue formazan. The absorbance of the cell lysates in dimethyl sulfoxide (Merk, Germany) solution was read at 495 ηm by Elisa Reader (Anthus 2020; England).

### RNA Extraction

All precaution procedures were applied in water and on laboratory instruments to inactivate RNase enzymes. Using the QIAGEN RNeasy mini kit (QIAGEN, Germany), total RNA was extracted according to the manufacturer’s protocol. The RNA quality was assessed using a 1.5% agarose gel and 18S/28S rRNA. Moreover, RNA quantity was analyzed using a NanoDrop 1000 (Thermo, USA) spectrophotometer.

### Quantitative Real-Time Polymerase Chain Reaction (PCR)

First, using RevertAid H Minus strand cDNA synthesis kit (Fermentase, Lithuania), strand cDNA was produced from total RNA (1μg). Then, specific primer pairs to amplify *BTG3* and *β-actin* (internal control) were designed by Primer3 software (Table-1) and synthesized by Bioneer Company (South Korea). PCR amplification of cDNA was performed in 20μl mixtures using SYBR® Premix Ex Taq ^TM^ (Takara, Japan) and real-time PCR (Corbett, Australia) with initial denaturation at 95°C for 5 seconds followed by 40 cycles including second denaturation at 95°C for 5 seconds, annealing at 59°C for 15 seconds as well as elongation at 60°C for 30 seconds. Finally, a melting curve analysis was conducted when PCR amplification was completed.

### Statistical Analysis

The formula of 2^˄−ΔΔCt^ was applied to calculate the relative changes in gene expression. ΔCt was obtained through C_t_ (*BTG3*) – C_t_ (*β-Actin*), where C_t_ demonstrates the threshold cycle number. Statistical analysis was conducted using Statistical Package for the Social Sciences 21.0 (SPSS Inc., Chicago, IL, USA), Microsoft Excel 2013 (Microsoft Corporation, USA), and GraphPad Prism 5.0 (GraphPad Software Inc, California, USA) software. A P<0.05 was considered as statistically significant. Additionally, Spearman and Mann-Withney tests were employed to investigate the pattern of target gene expression, growth rate, and others obtained continuous data.

## Results

**Figure 1:**
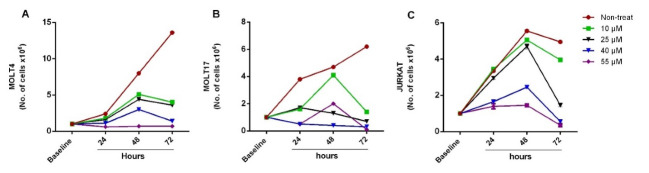
The number of viable cells of MOLT4 (**A**), MOLT17 (**B**), and JURKAT (**C**) cell lines, in the effect of various doses of genistein in three days of culture.

**Figure 2:**
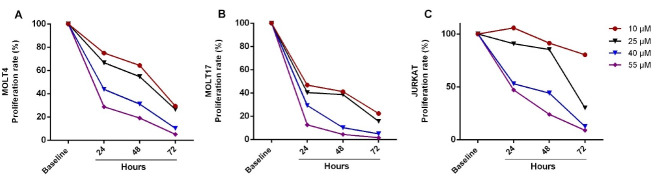
The normalized percentage of proliferation of MOLT4 (**A**), MOLT17 (**B**), and JURKAT (**C**) cell lines, respectively, compared to the non-treated group in terms of the effect of different doses of genistein in three days of culture.

**Figure 3:**
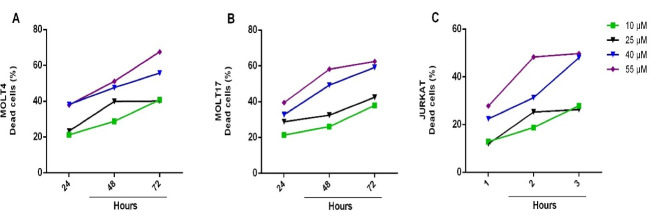
The normalized percentage of dead cells of MOLT4 (**A**), MOLT17 (**B**) and JURKAT (**C**) in terms of the effect of various doses of genistein compared to the non-treated group in three days of culture.

To evaluate the exposure doses of genistein, a 3-day surviving assay for ALL cell lines (MOLT4, MOLT17, and JURKAT) was conducted. The number of viable and dead cells was counted considering 24-hour intervals after treatment by single doses of 10, 25, 40, and 55μM genistein, and continued for 72 hours for which growth curves could be depicted (Figure-1). Proliferation rate was calculated as follow: the number of viable cells, which were treated with one of the genistein doses on each day was divided by the number of viable cells in the non-treated group on the same day of culture, and then were multiplied by 100 (Figure-2). According to the growth curve and its comparison with the non-treated group, 64, 41, and 91% of the proliferation occurred as the result of 10μM genistein effect on the second day of culture in case of MOLT4, MOLT17, and JURKAT cells, respectively. In other words, 35, 60, and 10% reduction rates were observed in the proliferation of MOLT4, MOLT17, and JURKAT cells resulting from the effect of 10μM genistein on the second day of culture. Whereas, 70, 90, and 60% reduction in proliferation of MOLT4, MOLT17, and JURKAT cells were observed after 48 hours treatment with 40 μM, respectively (Figure-2). No significant difference (P>0.05) in the reduction of proliferation rate was calculated between doses 40 and 55 μM. After 3 days of treatment with higher concentrations of genistein, the growth rate of MOLT4, MOLT17, and JURKAT cells were respectively reduced to 36, 10, and 26% as compared to untreated controls. It was also observed that the toxicity of genistein was more in MOLT17 in comparison to other ALL cell lines. The cytotoxic effect of different concentrations of genistein on three ALL cell lines during different periods was investigated using the MTT assay. The normal death rate of MOLT4 cells after being treated with 10μM genistein for 24, 48, and 72 hours in comparison to non-treated cells was increased to 21.2%, 28.8%, and 40.7%, respectively (Figure-3A). Almost the same trend was observed in concentrations of 25, 40, and 55 μM genistein and for MOLT17 (Figure-3B) and JURKAT (Figure-3C). The death rate more than 55% was observed in this respect. An increase in the rate of dead cells (including necrosis apoptosis) up to 67% on the third day of culture was observed in cell lines with increasing the concentration of genistein. It seems that genistein-induced anticancer proportion particularly anti-proliferative affectivity in a dose- and time-dependent manner. However, no significant difference was calculated between the concentration of 40 and 55μM in terms of reducing the growth and increasing the death rate of hematopoietic cancer cells.

### Expression of BTG3

**Figure 4:**
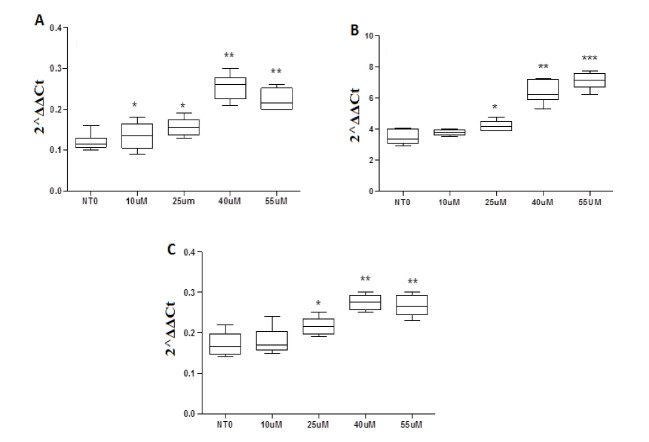
The Comparison of the relative fold (2^ΔΔCt) of *BTG3* in MOLT4 (**A**), MOLT17 (**B**), and JURKAT (**C**) cell lines in comparison to the non-treated group (NT0); after 48-hour treatment with various doses of genistein. * P≤0.01, **P≤ 0.001, ***P≤ 0.0001

To determine the effect of different concentrations of genistein on expression levels of BTG3 gene in three ALL cell lines, real-time PCR analysis was performed after 24 and 48 hours. The expression level of BTG3 gene in MOLT4 cells after being treated with 10 μM genistein for 24 and 48 hours in comparison to non-treated cells did not show a significant alteration, while the expression level was increased to 1.4, 2.7, and 1.65 folds after treatment with 25, 40, and 55μM genistein for 24 hours. Besides, the mRNA expression was increased up to 1.7 fold after 48 hours treatment with 40μM genistein (Figure-4). Interestingly, the expression of BTG3 slightly lessened after being exposed to 10μM genistein in 24- or 48-hour treatment. The expression level of BTG3 gene in MOLT17 cells after being treated with 10, 25, and 40μM genistein for 24 hours in comparison to non-treated cells was increased to 1.33, 2.15, and 1.51 folds, while 55μM of genistein did not show a significant alteration. Furthermore, the expression level after treatment with 10 and 25 μM genistein for 48 hours did not show a significant alteration; however, it was increased to 2.75 and 2.74 folds after treatment with 40 and 55μM genistein for 48 hours (Figure-4). The expression level of BTG3 gene in JURKAT cells after treatment with 10 and 25μM genistein for 24 hours in comparison to non-treated cells did not show a significant alteration, while the expression level was increased to 1.68 and 1.51 folds after treatment with 40 and 55μM of genistein. Up-regulation of BTG3 was significantly observed for 1.25, 1.65, and 1.49 fold after 48 hours treatment with single doses of 25, 40, and 55μM genistein.

## Discussion

Owing to the increased prevalence of ALL, efforts for finding new and novel therapeutic or preventive applications have increased worldwide in recent years [24]. Genistein, which is abundantly found in soybean, is a potent inhibitor of tyrosine kinase involved in proliferation signal cascades of cell growth [25], and moderate doses of genistein have been found to have inhibitory effects on the prostate, cervix, brain, breast, and colon cancers [26]. The present research was conducted to find out whether genistein has inhibitory impacts on the growth of leukemia cell lines or not. The second question was suggested to examine whether *BTG3* pathway was involved in the inhibitory effects of genistein on ALL cell invasion. Different action mechanisms are reported for genistein. One possible mechanism is the inhibition of topoisomerase II-decreasing cell proliferation [27]. It was observed that genistein could inhibit tumor cells in gastric, breast, lung, prostate, pancreatic, liver, ovarian, colon, and bladder cancer [28, 29]. Genistein not only prevents tumor cell growth, invasion, and metastasis but also increases tumor cells sensitivity to chemotherapy. Several studies have tried to reveal possible action mechanisms of genistein in gastric cancer [27, 30, 31]. In gastric cancer, genistein decreases Gli1 gene expression and attenuates cancer stem-like properties. Through this mechanism, genistein prevents invasion of tumor cells and inhibits tumor growth and metastasis [27]. Genistein also reduces gastric cancer chemo-resistance [30]. Another mechanism is increasing tumor suppression PTEN expression [32]. In addition to the above studies, some cohort studies have reported the decreasing risk of gastric cancer with isoflavones consumption [33]. Although the anti-cancer feature of genistein has already been noticed as a candidate herbal chemotherapeutic agent in different cancers, its effect in growth inhibition has been linearly related to genistein concentration. Carlo-Stella *et al.*, (1996) revealed that genistein strongly inhibited the growth of normal and leukemic hemopoietic progenitors; growth inhibition was dose- and time-dependent; leukemic progenitors were more sensitive than normal progenitors to genistein-induced growth inhibition [34]. Li *et al.,* (1999) reported the role of genistein in cell growth and apoptosis-related gene expression in breast cancer cells MDA-MB-231 [35]. They found the up-regulation of the expression level of Bax and p21^WAF1^ and down-regulation of Bcl2 and p53 in treated cells with genistein. They showed that apoptotic cell deaths occur via treating cells with genistein, and flow cytometry demonstrated that with the longer treatment of genistein, the number of apoptotic cells increased. They concluded that genistein had an inhibitory effect on the growth of MDA-MB-231 breast cancer cells, regulation of the expression of apoptosis-related genes, and induction of apoptosis through a p53-independent pathway. These findings suggest that genistein can be an effective chemopreventive and therapeutic agent against breast cancer [35]. Zhang *et al.,* (2008) demonstrated that the treatment of prostate cancer cells with low-dose genistein could be a strategy to suppress cell invasion through reversal of epithelial-mesenchymal transition (EMT), showing the potential use of genistein as a chemopreventive agent for the patients who have prostate cancer [36].Similarly, Howard *et al.,* (2006) showed that genistein displayed several biological activities leading to prostate cancer prevention, and this could be considered a cancer chemopreventive agent [37]. In addition, Severson *et al*., (1989) suggested genistein with an anti-metastatic activity for prostate cancer such that consumption of genistein led to lower incidence of clinical prostate cancer metastasis [38]. Several Studies demonstrated the role of genistein in the inhibition of cell growth (in both hormone-dependent & -independent cancer cells) in a dose-dependent manner [39-42]. Russo *et al.,* (2016) also demonstrated that genistein had an anti-proliferative activity in pharmacological doses (higher than 10 μM) which suggests that genistein might have in vivo anticancer effects [41]. It was also revealed that genistein directly inhibits Akt and *NF-κB* pathways (two important pathways in the activation of apoptosis) in prostate malignant cells [41, 42]. Weiqi *et al.,* (2013) investigated the action mechanism of genistein in hepatocellular carcinoma cells. They showed that genistein exhibited antitumor activity through modulating cellular motility and migration [43]. According to the available literature on the web, there is no documented study regarding the effect of genistein as a potent growth inhibitor against ALL, and it seems that it is the first study accomplished in this regard. Several studies have confirmed the positive relationship between soy isoflavones, specifically genistein, and cancer risk reduction [16, 22, 23, 43-54]. The present study investigated three ALL cell lines including MOLT17, MOLT4, and JURKAT that were treated with different concentrations of genistein (10, 25, 40, and 55 μM). MTT assay and cell counting were performed to analyze the cytotoxic effect of genistein and calculate the growth and normalized effective dose against malignant hematopoietic cells. According to our results, important correlations existed between genistein and reduced growth rate in all three cell lines, while suppressing the growth of MOLT4 was more as compared to other studied cell lines. Higher concentrations of genistein and longer treatment periods demonstrated more reduction in growth rate. Based on the results of the MTT assay, there was a direct proportion in line with increasing the percentage of dead cells, time, and dosage of exposure to genistein. The obtained data agreed with the results of other similar studies [51]. Our results confirmed the anti-proliferative effect of genistein that was correlated with the results of other similar investigations [11, 16, 21-23]. However, no significant difference was totally observed between 40 and 55uM. Therefore, the concentration of 40uM can be suggested to restrain or allay the growth of ALL cell lines.High correlation was observed between genistein and the growth reduction rate in a time- and dose-dependent manner. In other words, higher concentrations of genistein and longer treatment periods demonstrated more reduction in growth rate. Increasing the time of exposure to a higher non-toxic dose of genistein could effectively induce its anti-proliferative effect against acute lymphoblastic leukemia. Certainly, the reduction in growth rate in 72 hours could be due to the lack of nutrients in the cell. Although it seems that genistein induces its anti-cancer properties on all doses, a strong association indicated that these anti-cancer properties were negatively correlated with increasing the concentration and exposure time of genistein. Similar to the studies mentioned above, in the present study, we showed that genistein exhibited growth inhibitory property against leukemia cell lines. In fact, our findings were in line with those of other studies in terms of solid tumors. Lack of normal cells and detection the rate of early and late apoptosis due to no access to flow cytometry device can be considered as a limitation in the present study.Down-regulation of *BTG3* gene has been confirmed in several cancers [11-14, 16, 19, 20, 22, 48]. It was reported that increasing the expression of this protein deteriorated cell cycle progress from the G0 or G1 to S [52] particularly through the CCND1 pathway [53]. To the best of our knowledge, there is no information about *BTG3* expression levels in ALL diseases. According to the previous studies, the overexpression of *BTG3* using an expression vector can cause a significant decrease in the proliferation rate in cancer cell lines [18]. Of course, determine the protein level due to project financial constraints, can also be counted as another limitation of this study. Here, we, for the first time, examined the time-response and dose-response of BTG3 expression in ALL cell lines treated with genistein using quantitative RT-PCR. Few studies have remarked that *BTG3* expression can be restored by genistein similar to what 5-aza-2′-deoxycytidine can induce [11, 16, 54]. The results directed that treatment with 25, 40, and 55uM of genistein caused up-regulation of *BTG3* in a dose- and time-dependent manner in these examined ALL cell lines. Therefore, it can be deduced that one of the pathways for anti-cancer action of genistein is through *BTG3* as a tumor suppressor gene with anti-proliferative properties.

## Conclusion

According to the findings, genistein, as a natural and biocompatible compound, can act as an anti-proliferative and consequently retardant agent against the development of ALL cancer cells similar to solid tumor cells. Concerning the expression increase of *BTG3* as a tumor suppressor gene, what it is pioneering in this study is to suggest another action mechanism of genistein. Therefore, it can be concluded that this natural isoflavone could be considered as an agent to potentially control the invasion of leukemia cells and expedition of disease, which is promising and fascinating.

## Acknowledgment

This project was financially supported by the Research Vice Chancellor of Hormozgan University of Medical Sciences (HUMS), Bandar Abbas, Iran, under grant No. 24/MTF/292.

## Conflict of Interest

There is not any conflict of interest regarding this study and the authors. Additionally, this article does not involve any study that has used human participants or animals.

**Table 1:** Primers Designed for Amplification of BTG3 and β-Actin.

**Gene**	**Primer sequence (5’→3’)**	**Product size (bp)**
***BTG3***	F: GTAAGGAAATGGAAGTGAAACC	176
	R: AATGGAACAGGAGGAGGAT	
***β*** ***-*** ***Actin***	F: GCCTTTGCCGATCCGC	90
	R: GCCGTAGCCGTTGTCG
